# Supplementary material for: Testing an analogue game to promote peer support and person‐centredness in education for people with diabetes: A realist evaluation
Source: Nurs Open. 2021 Mar 2;8(5):2536–50. doi: 10.1002/nop2.784 (PMC8363400; doi:10.1002/nop2.784)
Supplement: Supplementary file 1 — App S1 [file NOP2-8-2536-s002.docx]

| **Purpose** | **Activities**  **Appendix S1** Overview of the analogue game development process | **Participants** |
| --- | --- | --- |
| Phase 1: Ideation  Define the approach and learning objectives | Kick-off workshop for brainstorming ideas  Interviews with domain stakeholders and specialists to generate ideas | Researchers and game designers  Diabetologist and diabetes nurse |
| Develop the core game structure  Develop early concept prototypes | Game-design workshop to generate ideas  Interactive co-creation workshops focusing on themes and methods for the game guided by principles to promote person‑centredness and peer support  Developing and iterating on the game structure, narrative and content using patient quotes and personas based on existing educational material | Game designers  Researchers and game designers  Researchers and game designers |
| Phase 2: Development  Iteratively prototype, test and redesign the game based on observations and user feedback | Fine-tuning the game structure  Exploring reactions to preliminary prototypes from stakeholders by testing and adjusting prototypes in eight DSME sessions  Adjusting prototypes based on user feedback, (e.g., developing a new point system, adding more pictures and condensing the text)  One test with external game designers and two tests with researchers | Game designers  1-2 HCPs and 3-12 PWT2D per test from eight different settings across three municipalities  Game designers and researchers |
| Finalize graphic design, enhance usability and initiate manual production | Final workshops with researchers and game developers  Writing the facilitator manual  Developing graphic design  Testing the game with researchers and game developers  Making final adjustments to contents and graphic design | Researchers and game developers  Researchers  Graphic designer  Researchers and diabetes psychologist  Researchers and game designers |
