# Supplementary material for: Testing an analogue game to promote peer support and person‐centredness in education for people with diabetes: A realist evaluation
Source: Nurs Open. 2021 Mar 2;8(5):2536–50. doi: 10.1002/nop2.784 (PMC8363400; doi:10.1002/nop2.784)
Supplement: Supplementary file 2 — App S2 [file NOP2-8-2536-s001.docx]

**Appendix S2** Questionnaire content

| **Item** | | | **Question (PWT2D or/and HCP)** | **Response categories** |
| --- | --- | --- | --- | --- |
| ***Items on participant characteristics*** | | | | |
| Gender | | | What is your gender? (PWT2D/HCP) | 1. Female 2. Male 3. Other   (only one answer) |
| Age | | | What is your age? (PWT2D/HCP) | _______ years |
| T2D duration | | | How long have you known that you had diabetes? (PWT2D) | _______ years |
| T2D treatment | | | Current medical treatment for your diabetes? (PWT2D) | 1. Insulin 2. Oral medication for high blood sugar   (multiple responses possible) |
| Comorbidities | | | Do you have other chronic illnesses? (PWT2D) | 1. Yes 2. No 3. If yes, please describe _______ |
| Educational level | | | What is your educational level? (PWT2D) | 1. Primary or lower secondary 2. Upper secondary 3. Vocational training 4. Short post-secondary education (< 3 years) 5. Post-secondary education (≥ 3 years) 6. University post-secondary education (≥ 5 years)   (only one answer) |
| Cohabitate status | | | Do you live with others? (PWT2D) | 1. Spouse / cohabitant / boyfriend? 2. Home living children   (multiple responses possible) |
| HCP Experience working with PWT2D | | | How long time have you worked as a diabetes educator? (HCP) | Open-ended response |
| HCP profession | | | What is your profession?  (HCP) | Open-ended response |
| ***Measure of overall experience of the board game*** | | | | |
| Overall perception of the analogue game | | | Overall, what is your perception of the board game? (PWT2D/HCP) | The items were rated on a five-point Likert-type scale ranging from:   1. Excellent 2. Very good 3. Good 4. Lees good 5. Bad   (only one answer) |
| ***Measures to assess perception of the game structure*** | | | | |
| Perception of the game structure | The following questions is about your perception of the game structure. We would like you to assess the degree to each of them:   1. Game topics were relevant to everyday life with T2D (PWT2D/HCP) 2. The point system was easy to understand (PWT2D/HCP) 3. The game rules were easy to understand (PWT2D/HCP) 4. Fictitious persona seemed authentic   (PWT2D/HCP)   1. The game was fun to play (PWT2D/HCP) 2. Playing the fictitious persona made me think of own life with T2D (PWT2D) 3. The persona made PWT2D think of their own lives with T2D (HCP) | | | The items were rated on a five-point Likert-type scale ranging from:   1. Highly agree 2. Agree 3. Neither agree nor disagree 4. Disagree 5. Highly disagree   (only one answer) |
| ***Measures to assess perception of dialogue and active engagement*** | | | | |
| Perception of dialogue and active engagement | | The following questions is about your perception of the dialogue while playing the board game. We would like you to assess the degree to each of them:   1. Talked well together during the game (PWT2D/HCP) 2. Talked with others about diabetes (PWT2D/HCP) 3. Listened to each other during the game (PWT2D/HCP) 4. Heard about other experience with diabetes (PWT2D/HCP) 5. Experienced a sense of collectiveness with others who have diabetes (PWT2D/HCP) 6. Experienced a good atmosphere (HCP) 7. The game encouraged dialogue within the group (HCP) 8. Playing the game ensured that everyone got a chance to talk (HCP) 9. Encouraged the PWT2D to talk more than usual in the DSME (HCP)   On a scale from 1 to 10, to which degree did the game promoted useful knowledge on the experience and view on diabetes of each participant with T2D (HCP) | | The items were rated on a five-point Likert-type scale ranging from:   1. Highly agree 2. Agree 3. Neither agree nor disagree 4. Disagree 5. Highly disagree   (only one answer)  (1 = no degree of new knowledge, 10 = high degree of new knowledge) |
| ***Measures to assess HCPs perception of implementing the analogue game in future work*** | | | | |
| Implementing the analogue game in future work | | On a scale from 1 to 10, how likely is it that you will use the analogue game in my future work (HCP)  On a scale from 1 to 10, how likely is it that you will recommend the game to a colleague (HCP) | | (1 = not likely, 10 = very likely) |
